# Locus suicide recombination actively occurs on the functionally rearranged IgH allele in B-cells from inflamed human lymphoid tissues

**DOI:** 10.1371/journal.pgen.1007721

**Published:** 2019-06-14

**Authors:** Iman Dalloul, François Boyer, Zeinab Dalloul, Amandine Pignarre, Gersende Caron, Thierry Fest, Fabrice Chatonnet, Céline Delaloy, Anne Durandy, Robin Jeannet, Emilie Lereclus, Hend Boutouil, Jean-Claude Aldigier, Sophie Péron, Sandrine Le Noir, Jeanne Cook-Moreau, Michel Cogné

**Affiliations:** 1 CNRS UMR 7276 / INSERM U1262, Université de Limoges, Limoges, France; 2 INSERM U1236, Université de Rennes; EFS Bretagne, Rennes, France; 3 Institut Imagine INSERM U1163, Paris, France; 4 MC and JCM co-directed this work; University of Washington School of Medicine, UNITED STATES

## Abstract

B-cell activation yields abundant cell death in parallel to clonal amplification and remodeling of immunoglobulin (Ig) genes by activation-induced deaminase (AID). AID promotes affinity maturation of Ig variable regions and class switch recombination (CSR) in mature B lymphocytes. In the IgH locus, these processes are under control of the 3’ regulatory region (3’RR) super-enhancer, a region demonstrated in the mouse to be both transcribed and itself targeted by AID-mediated recombination. Alternatively to CSR, IgH deletions joining Sμ to “like-switch” DNA repeats that flank the 3’ super-enhancer can thus accomplish so-called “locus suicide recombination” (LSR) in mouse B-cells. Using an optimized LSR-seq high throughput method, we now show that AID-mediated LSR is evolutionarily conserved and also actively occurs in humans, providing an activation-induced cell death pathway in multiple conditions of B-cell activation. LSR either focuses on the functional IgH allele or is bi-allelic, and its signature is mainly detected when LSR is ongoing while it vanishes from fully differentiated plasma cells or from “resting” blood memory B-cells. Highly diversified breakpoints are distributed either within the upstream (3’RR1) or downstream (3’RR2) copies of the IgH 3’ super-enhancer and all conditions activating CSR *in vitro* also seem to trigger LSR although TLR ligation appeared the most efficient. Molecular analysis of breakpoints and junctions confirms that LSR is AID-dependent and reveals junctional sequences somehow similar to CSR junctions but with increased usage of microhomologies.

## Author summary

Class switch recombination, initiated by the activation-induced deaminase enzyme rearranges immunoglobulin (Ig) genes in order to replace expression of IgM by IgG, IgA or IgE. A variant form of this event, locus suicide recombination (LSR), was previously reported in mouse B-lymphocytes and simply deletes all functional Ig constant genes, thus terminating B-cell function. This study first demonstrates that the structure of the human Ig heavy chain locus provides an ideal target for LSR, and is thus actively (but transiently) affected by this deletion process at the activated B-cell stage. LSR then yields recombined genes that do not support B-cell survival and which thus becomes almost undetectable among long-lived memory B-cells or plasma cells.

## Introduction

Humoral immune responses and immunoglobulin (Ig) production rely on the selection of B-cells harboring antigen (Ag)-specific B-cell receptors (BCRs). This selection implies not only proliferation and differentiation of those cells optimally binding Ag but also elimination of the less efficient or inappropriately activated cells. The latter can be accomplished through various pathways leading to anergy, death-by-neglect or activation-induced cell death (AICD). While AICD pathways have been characterized in detail for T-cells, and notably involve FAS-induced apoptosis, they are less documented in B-cells. A major and unique feature of mature B-cells during Ag-driven responses, is their ability to reshape their genome, and more specifically Ig genes, after activation-induced deaminase (AID)-dependent modifications. Somatic hypermutation (SHM) within germinal centers (GC) can yield B cells with higher affinity V domains, which preferentially capture Ag from follicular dendritic cells, undergo stimulatory cognate interactions with T follicular helper cells and are further selected for survival. In parallel, AID-dependent class switch recombination (CSR) diversifies IgH classes by joining repetitive switch (S) regions that precede the various constant (C) genes. Besides the selected winners of AID-mediated reshaping, many cells are losers or undesired responders deserving elimination, in agreement with the considerable amount of GC B-cells which have been demonstrated to be actively undergoing apoptosis [1,2]. Out-of-frame or other unfavorable V region mutations might result in BCR loss and promote apoptosis while more subtle cell fate decisions will arbitrate between death, short-term or long-term survival as memory lymphocytes or plasma cells [1,3]. Such intra-GC cell fate choices are crucial since inappropriate survival or terminal differentiation of bystander cells producing useless Ig or Ig with increased affinity for self or environmental Ags might trigger auto-immunity, inflammation and disease. Since class-switched antibodies are potent actors of auto-immunity and/or hypersensitivity, means for restricting CSR and reentry of class-switched cells into SHM thus appear as necessary safeguards to keep humoral immune responses both specific and innocuous. The AID-dependent process of locus suicide recombination (LSR) reported in mouse B-cells ideally fits this necessity [4]. We now show that the human IgH 3’RR super-enhancers include sequences ideally suited as recombination targets, and that LSR actively occurs in human lymphoid B-cells.

LSR features recombination between Sμ and the 3’ regulatory region (3’RR) located downstream of the IgH locus, thus deleting the whole IgH constant gene cluster. The 3’RR includes several B-lineage specific enhancers (hs3, hs1-2 and hs4) which are mostly active after B-cell stimulation and which then promote transcription and recombination [5]. Targeted mutations of the 3’RR in the mouse demonstrated its major role in SHM, its control of germline transcription and CSR to most C genes, its booster effect on IgH gene expression in plasma cells and even its ability to promote inter-allelic transvection between IgH alleles [5–11]. The mouse 3’RR has a unique palindromic architecture with functional implications [12–15], and this structure is shared by all mammalian species studied to date, including humans [16]. In addition, the mouse 3’ RR includes multiple stretches of repetitive DNA resembling S-regions albeit shorter [4,12], potentially promoting LSR through a mechanistic process similar to CSR. In B-cells, Ag stimulation with appropriate co-stimuli induces AID expression which then initiates staggered DNA nicking in repetitive S-regions, double strand breaks (DSBs) and CSR. Highly repetitive “Like switch” (LS) regions from the 3’RR might serve a similar role, ending with Sμ-3’RR junctions and deletion of the entire IgH constant region gene cluster. By abrogating BCR expression, which is critical for B-cell survival, this eliminates the corresponding cells.

In humans and old-world great apes, an internal duplication of the IgH locus constant gene cluster has additionally duplicated 3’ regulatory regions downstream of each Cα gene (3’RR1 and 3’RR2 respectively downstream of Cα1 and Cα2)[16,17]. We show that both human 3’RRs include LS-regions which are highly structured in terms of DNA repeats and which can locally be G-rich, with predicted ability to form G-quadruplex (G4) DNA now known as the ideal substrate for AID [18,19]. Importantly, these regions also actively undergo AID-dependent recombination in activated B-cells both *in vivo* and *in vitro*, regardless of the activation conditions assayed.

## Results

### Human 3’RR1 and 3’RR2 share multiple common features with S regions

**Fig 1:**
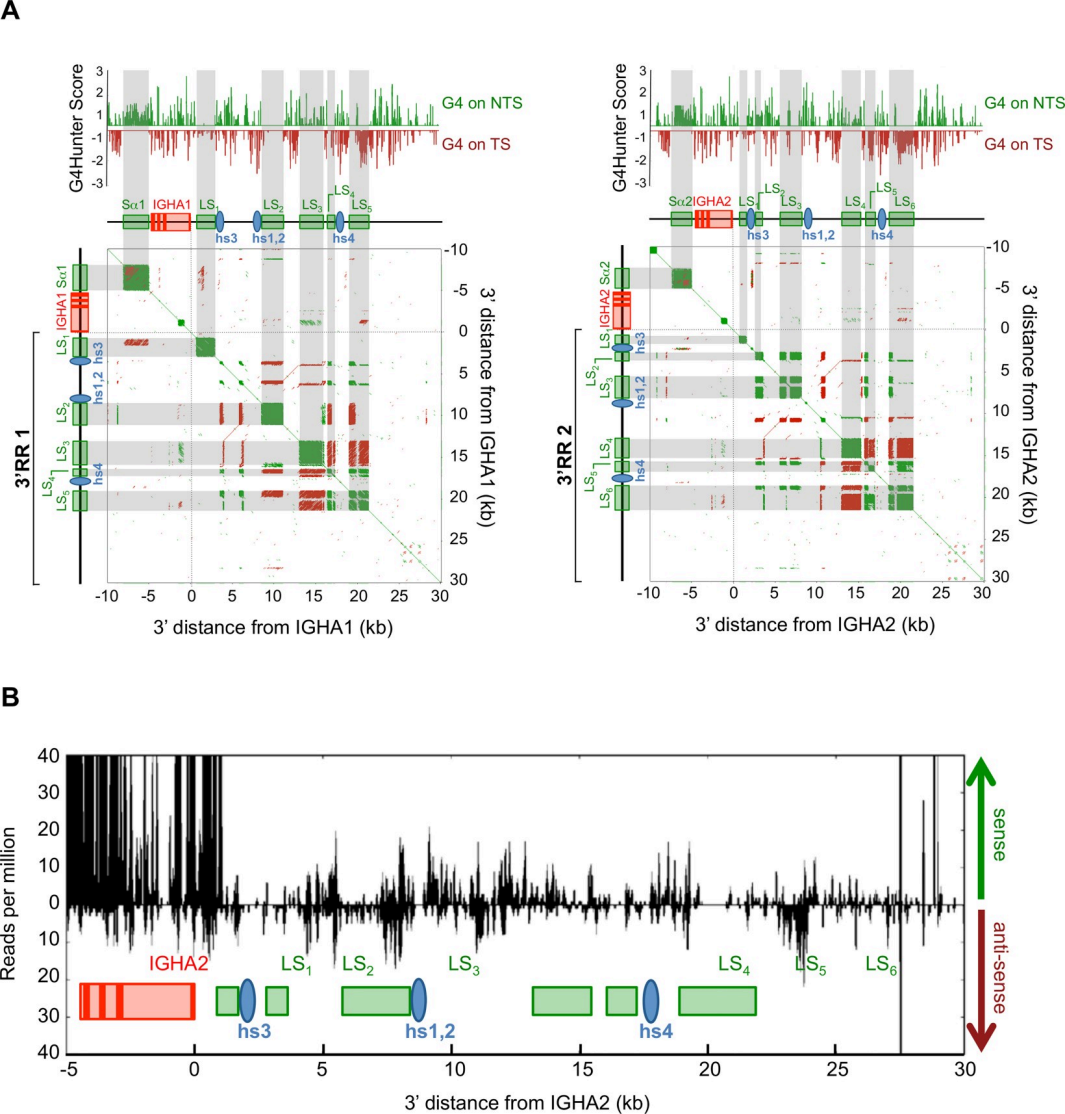
3’RR structure and transcription in activated B-cells. (A) Structure of genomic fragments including either human Sα1, Cα1 gene and 3’RR1 (left), or Sα2, Cα2 and 3’RR2 (right) was analyzed for presence of G4 DNA on the template (TS) or non-template (NTS) strand (top). Dot plot analysis of internal repeats (bottom) was run with the YASS algorithm: colored dots indicate short stretches of homology in direct (green) or inverted (red) orientation (green squares thus mark positions of highly repetitive S or LS regions / red lines indicate positions of inverted repeats, notably the palindrome centered on the hs1,2 enhancer). (B**)** Transcription of human 3’RR2 in sense and antisense orientation, as evaluated by RNA-seq experiments with RNA from *in vitro* activated B-cells.

Architectures of human 3’RR1 and 3’RR2 elements were analyzed for the presence of direct and inverted repeats (Fig 1). As previously described for the mouse 3’RR, “like-switch” (LS) sequences (*i*.*e*. stretches of highly repetitive DNA resembling S regions) were readily evidenced using the YASS dot plot algorithm (http://bioinfo.lifl.fr/yass/yass.php) for a survey of direct repeats in a window range set over 20bp, with a minimal identity threshold fixed at 90% (Fig 1A). Five to six human LS regions stand in each of the human IgH 3’RR super-enhancers. These LS regions are each 0.5 to 2 kb long, and are interspersed with core enhancer elements, which they do not overlap.

Human 3’RR LS sequences additionally show 35–50% G-richness on one or the other DNA strand, and analysis using the G4-hunter algorithm http://bioinformatics.cruk.cam.ac.uk/G4Hunter/ [20], predicted the occurrence of several clusters of G4-DNA, on either one or the other DNA strands of LS regions (Fig 1A).

When checking RNA-seq data from activated human B-cells, we found that 3’RR transcription occurred in both the sense and antisense orientations in reference to IgH constant genes, and that, quantitatively, it revealed equivalent amounts of transcripts corresponding to either orientation (Fig 1B). This is in contrast to IgH constant genes (see for example Cα and the preceding Sα region on Fig 1B), which are predominantly transcribed in the sense orientation.

### LSR occurs at multiple sites within the whole extent of both human 3’RRs

**Fig 2:**
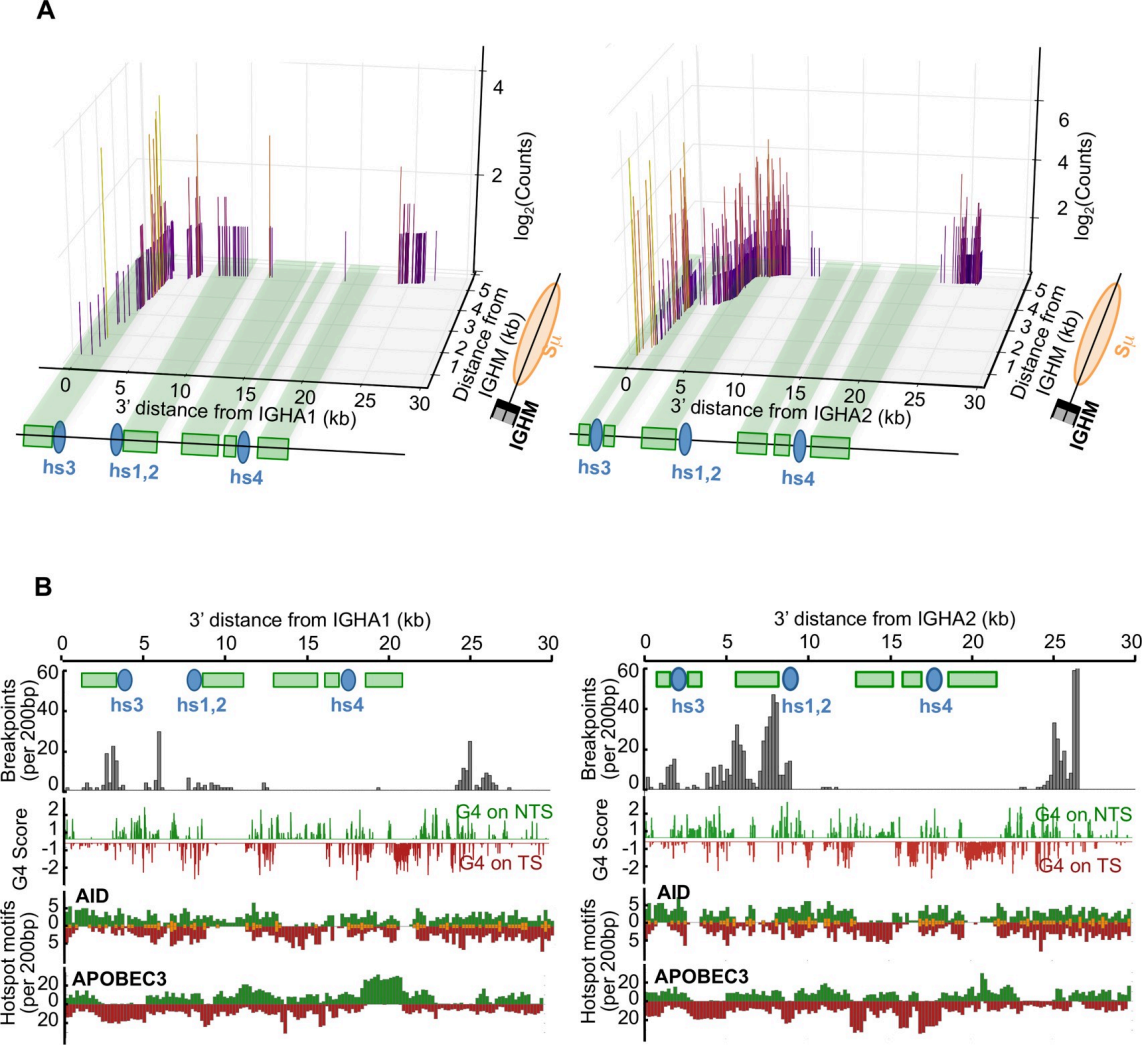
Positions of LSR breaks in human activated B-cells. (A) 3D-schematic showing the positions of junctions sequenced in this study with regards to their abundance (in log scale) and position of DSBs in Sμ, and 3’RR1 (left) or 3’RR2 (right). (B) Maps of 3’RR1(left) and 3’RR2 (right) reporting breaks observed in sequenced LSR junctions together with the position of G4 DNA on either the non-template (green) or template (red) strand (with reference to the transcriptional orientation of IgH constant genes), AID consensus target sites, and APOBEC3 family deaminases.

Mouse LSR was initially characterized using nested PCR on DNA from B-cell samples, followed by specific hybridization [4]. In order to improve the specificity of the read-out for PCR assays on human samples, the final hybridization step of PCR products, was replaced in this study by an “LSR-seq” procedure with high-throughput sequencing and junctional sequence analysis adapted from our recently described CSR evaluation algorithm, CSReport [21]. The algorithm was adapted in order to consider 3’RR sequences as surrogate acceptor S regions and sequence determination thus precisely mapped the breakpoint sites. CSReport mapped LSR junctions linking Sμ to either the human 3’RR1 downstream of Cα1 or 3’RR2 downstream of Cα2 (Fig 2).

DNA amplification over a variable distance (ranging from hundreds of base pairs to more than 30kb, depending upon the position of the junctions) might preferentially amplify the shorter fragments. Despite this potential bias underestimating the occurrence of the most distant junctions, the combined use of prolonged elongation steps during PCR and of 3 primers located either downstream from the hs3, hs1,2 and hs4 enhancers resulted in the mapping of breakpoints located throughout 3’RR1 and 3’RR2 (Fig 2). The 3’RR breaks were joined to various positions within Sμ, with preferential use of breakpoints at the 5’ side of Sμ, especially for junctions with the downstream regions of the 3’RRs. When considering the sum of all junctions mapped in this study (Fig 2), the most striking observation was their high diversity and scattered distribution throughout the 3’RRs (both within LS regions and in their proximity).

It is questionable whether LSR targets only unswitched B-cells, thereby yielding direct Sμ-3’RR junctions, or if it can also target class-switched cells and then eventually conserve a remnant of a downstream S region inserted in-between Sμ and a recombined 3’RR. We indeed obtained multiple evidence of such “post-switch” LSR, with sequences obtained from the LSR-seq protocol (*i*.*e*. sequencing an Sμ to 3’RR amplicon) but including junctions of Sμ with Sγ or Sα, (or Sγ or Sα joined with a 3’RR). Out of a total of 2342 unique LSR junctions sequenced in this study, 343, *i*.*e*. about 14%, could be typed as complex junctions involving breaks with a downstream S (Sγ or Sα) region and certifying their origin from switched cells. This figure could be underestimated since “direct” Sμ-3’RR junctions are ambiguous: they could occur directly in IgM+ cells, or secondarily in switched cells, after deletions eliminating any intermediate Sγ or Sα remnant.

### LSR-seq can semi-quantitatively estimate *in vivo* LSR in freshly collected B-cells

CSR junctions were characterized in parallel to LSR junctions, by a similar PCR/sequencing strategy in order to provide an LSR/CSR comparison. “LSR-seq” and “CSR-seq” protocols thus provided a semi-quantitative assessment of recombination events with the number of sequences including the expected junctions. This evaluation eliminated all identical reads, in order to neutralize the effect of clonal size and potential biases in PCR amplification, thus simply considering the numbers of unique junction sequences. Although we cannot claim such a nested PCR and sequencing assay to be fully quantitative, it appeared to be at least semi-quantitative, giving negative results in non-B-cell samples, abundant signals in B-cells from chronically inflamed lymphoid tissues and progressively decreasing signals in successive dilutions of positive DNA (S1 Fig). It also had the abovementioned advantage of precisely characterizing sequencing reads with LSR (or CSR) junctions, and assigning redundant reads to the same unique junction.

**Fig 3:**
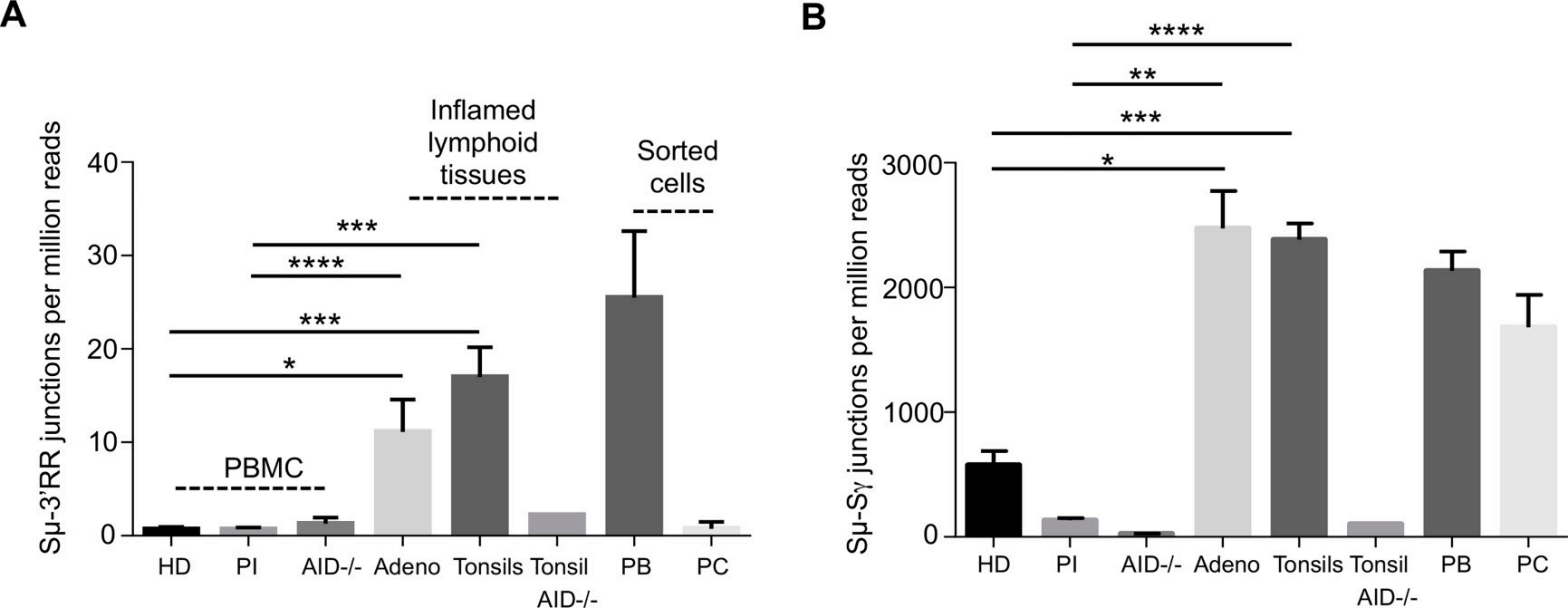
LSR breaks *in-vivo* in humans. (A) Sμ-3’RR LSR junctions were analyzed in peripheral blood B cells isolated from healthy donors (HD) (n = 6) but also in blood from recently hospitalized post-infection (PI) patients treated for bacterial infections (n = 17) and from AID-/- B-cells (n = 3). LSR was also studied using DNA from inflamed lymphoid tissues (adenoids (n = 3) and tonsils (n = 11)), 1 AID-/- tonsil, sorted plasmablasts (PB) isolated from tonsils (n = 5) and finally in DNA from terminally differentiated plasma cells (PC) (n = 2) sorted from bone marrow. Frequencies of LSR junctions amplified with an Sμ forward primer and a downstream hs4 reverse primer were evaluated in parallel to those of classical Sμ-Sγ CSR junctions (B) amplified from the same DNA samples, in order to provide an LSR/CSR comparison. Data represent mean numbers (junctions per one million reads) ± SEM. *P < 0.05, **P < 0.01, ***P < 0.001, ****P < 0.0001.

LSR junctions from Sμ to either 3’RR were accordingly semi-quantified in DNA from fresh primary B-cells from various origins. No LSR junctions were found in DNA from either tonsil B-cells nor PBMC obtained from AID-deficient patients (Fig 3). By contrast, in tissues from AID-proficient individuals, LSR was abundant in DNA from tonsils or adenoids. Peripheral blood mononuclear cells (PBMC) from healthy donors regularly yielded low, close to negative, LSR signals, and the same was observed in a series of patients recently treated for bacterial infections. In healthy donors, as expected from the weak signals given by PBMC, LSR was also absent or very low in sorted [CD19+, CD27+] peripheral blood memory B-cells and also in sorted bone marrow CD138+ plasma cells (Fig 3). Altogether, LSR junctions were thus rare in DNA from resting cells, either circulating B-cells, sorted memory B-cells or differentiated plasma cells and mostly detected in lymphoid tissues with active immune responses such as tonsils. Further splitting and sorting of tonsil cells showed that LSR junctions could be found at the highest level in cells with ongoing activation (sorted as blasting CD19+IgD-CD10-CD38^hi^ B-cells) (PB; Fig 3).

### LSR actively occurs *in vitro* upon B-cell activation

**Fig 4:**
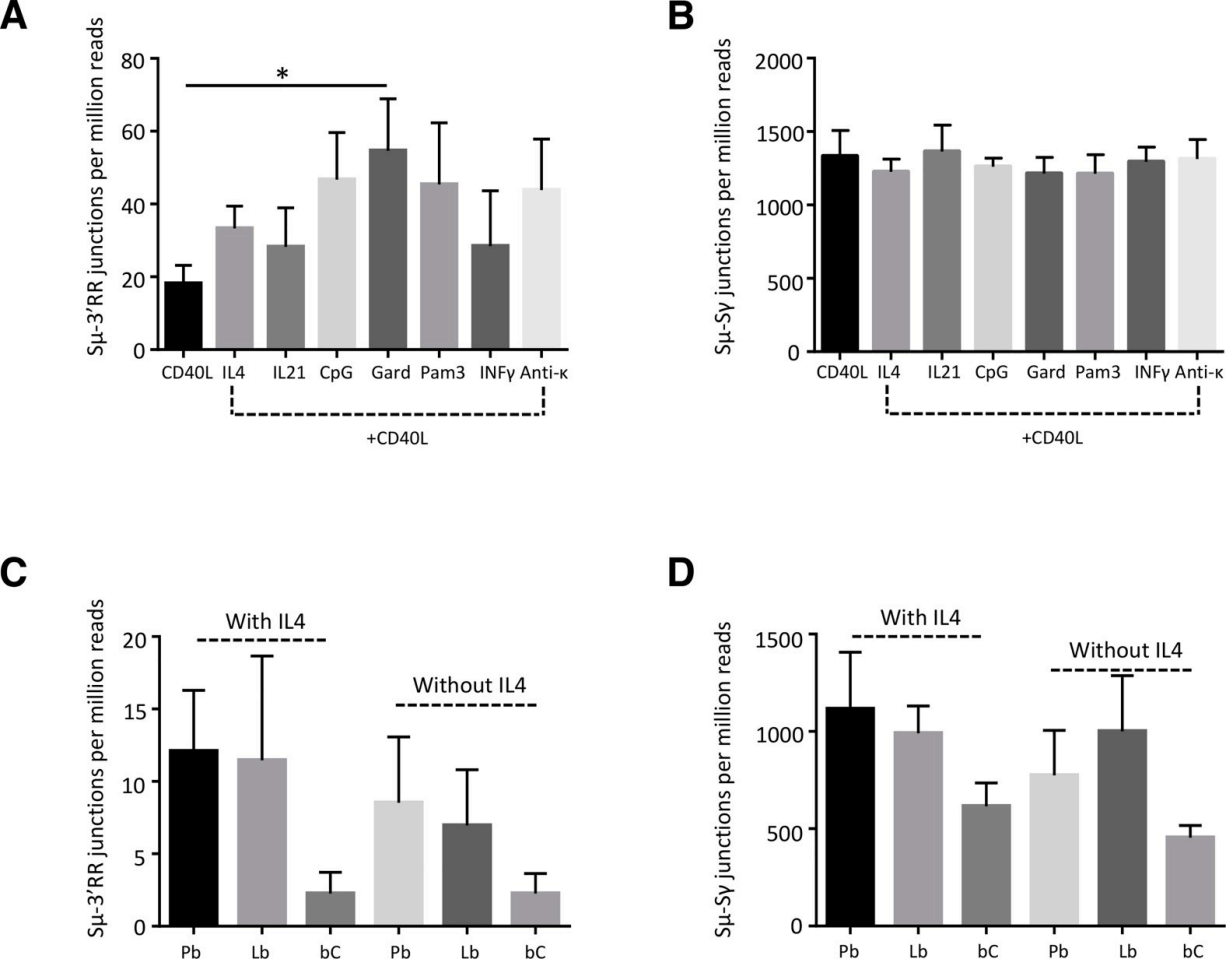
LSR after *in-vitro* stimulation. (A) Sorted B cells from peripheral blood B cells from healthy donors were activated for 4 days with human recombinant CD40L alone or with recombinant human IL4, IL21, CpG, gardiquimod (Gard), Pam3, IFNγ or with an anti-Igκ antiserum. LSR junctions were amplified using reverse primers located in hs3, hs1,2 and downstream hs4. Equal amounts of the three PCR products were combined in a single library. (B) IgG CSR junctions using the same stimulation conditions. Data represent means (for unique junctions per one million reads) ± SEM for 6 people. (C) LSR junctions in *in vitro* stimulated naive B-cells, sorted after stimulation. PBMC from healthy donors were activated *in-vitro* for 6 days with combined anti-CD40, TLR9 ligand CpG, anti-BCR antibodies and other cytokines generating three different stages of differentially activated- B-cells: Pb (plasmablasts), ABC (proliferating activated B-cells) and bC (bystander B-cells). LSR junctions were amplified using a reverse primer located in downstream hs4. Data represent mean number of junctions per million reads ± SEM for 3 donors. (D) CSR junctions to Sγ in same samples as C. CSR junctions were amplified using a downstream IgG consensus primer located in CH1. Data represent means (for unique junctions per one million reads) ± SEM.

Class-switching and induction of AID in human B-cells is mainly a T-dependent process and is most often modelled *in vitro* by activating B-cells with CD40 ligands. When sorted blood B-cells from healthy patients were stimulated in such conditions, LSR was induced simultaneously with CSR. Associating anti-CD40 stimulation with various co-stimulatory factors, increased LSR and the LSR/CSR ratio in most of the conditions assayed and notably upon addition of IL4, IL21, or BCR ligation with anti-κ antibodies, or, to the highest extent, upon TLR7 ligation by gardiquimod (Fig 4 A).

In order to more precisely identify those cells showing LSR junctions *in vitro*, we sorted three different activated B-cell populations, after a 6-day activation protocol combining anti-CD40, TLR9 ligand CpG, anti-BCR antibodies and cytokines, previously described as optimal for mimicking the *in vivo* humoral immune response [22]. This generated either plasmablasts committed towards plasma cell differentiation, or activated B-cells having proliferated but still at an earlier stage (resembling proliferating centroblasts), which were compared to surviving but non-dividing bystander B-cells from the same culture. Analysis of these three types of cells showed that LSR was virtually absent in non-dividing bystander B-cells, but was, by contrast, at the highest level in plasmablasts and at an intermediate level in actively proliferating activated B-cells (Fig 4C).

### Structure of LSR junctions

**Fig 5:**
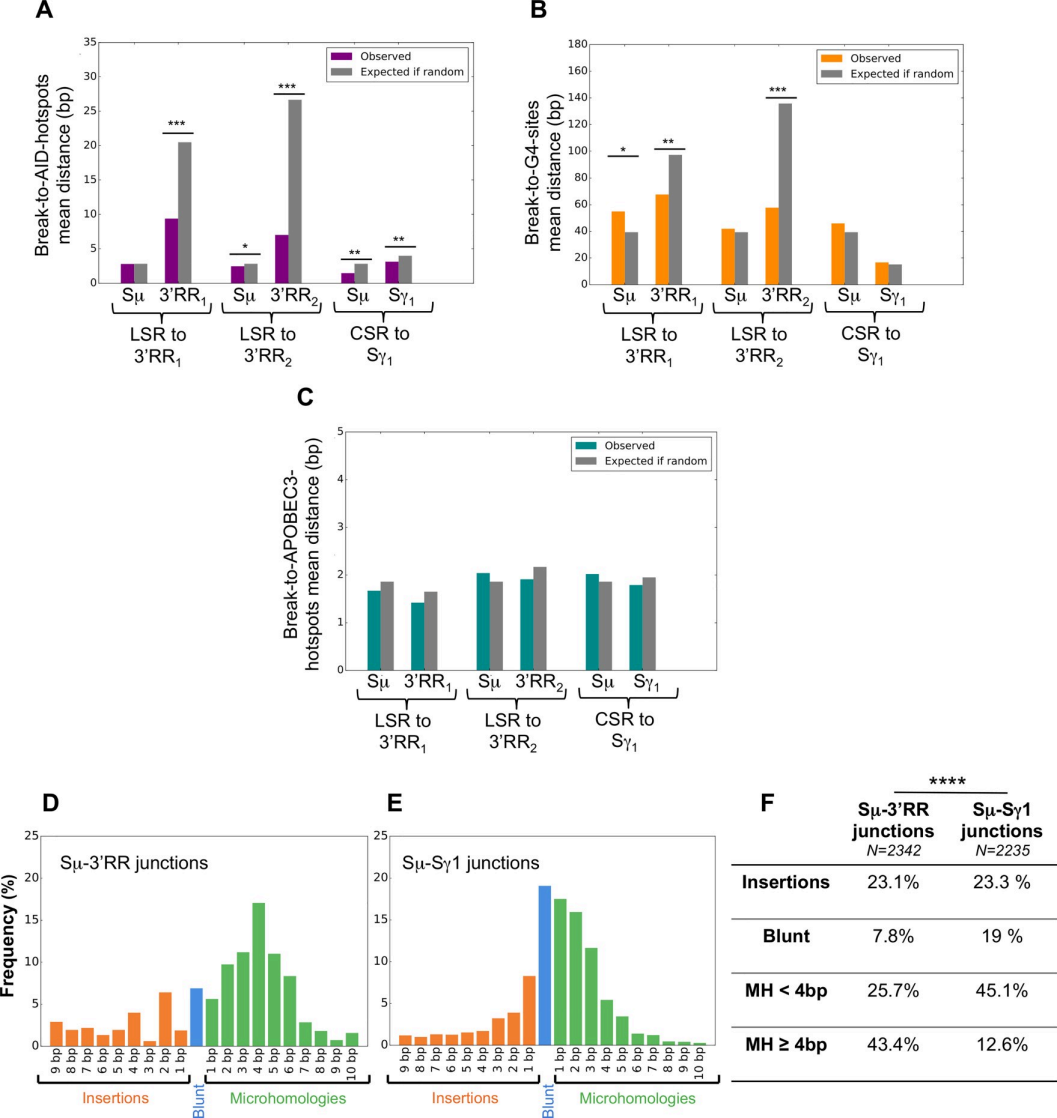
Detailed position of breaks and structure of human repaired LSR junctions. Mean distance to AID target sites (A), G-quadruplex (B) and APOBEC3 family (C) sites. Structure of repaired junctions with regards to mean number of inserted nucleotides and length of microhomologies between broken ends for Sμ-3’RR (D), Sμ-Sγ1 (E). Comparison of LSR vs CSR junction structure in percentage (F).

CSR junctions from Sμ to Sγ and LSR junctions from Sμ to 3’RR were amplified by PCR with specific primers, sequenced by NGS (Ion Proton) and analyzed regarding their positions in S regions and/ or the 3’RR, and their nature using CSReport, a dedicated tool that performs junction assembly and analysis based on BLAST alignments (Fig 5).

A first difference between LSR and CSR concerned the global position with regard to repetitive regions. All Sμ breaks were located in the repetitive part of Sμ (in agreement with the fact that in the mouse, deletion of Sμ repeats virtually abrogates CSR)[23]. By contrast, in the 3’RRs, breaks were found not only within but also outside of LS regions, notably downstream of hs4 (Fig 2).

We explored whether the sequences of junctions provided clues into the likely mechanisms of DNA breakage. We thus scored the position of each DNA donor and acceptor break involved in a sequenced junction and its distance to the genomic features relevant to DNA cleavage: preferential AID target sites (WRCY / RGYW), APOBEC3 target sites (TC / GA) and G4 sites (as suggested by the G4 Hunter algorithm) (Fig 5A–5C). This observed distance to the closest target sequence was compared to the calculated distance expected if a random break occurred in the same region. In the 3’RR, observed break to AID site distances were much shorter than expected distances if random breaks were occurring, thus emphasizing the role of AID in the initiation of LSR acceptor breaks. The same was true for G4 sites within the 3’RR, the proximity of which also seemed to favor DNA breakage. For LSR as well as CSR donor breaks located within Sμ, both observed break to AID site and break to G4 site distances were very low, due to the highly repetitive structure of S-regions carrying abundant AID target sites and G4-rich DNA. Even distances calculated under the hypothesis of random breaks then appeared by force very low (Fig 5). The same analysis for APOBEC3 family target sequences showed no difference between the distances observed and those calculated under the hypothesis of random breaks, neither in Sμ nor within the 3’RR, making it unlikely that APOBEC enzymes significantly contribute to DNA breakage (Fig 5C).

LSR junctional sequences differed from CSR in their classification by the CSReport algorithm as either blunt, (broken ends directly joined without overlap), microhomologous (with stretches of a few nucleotides shared between joined segments) or with insertions (a few nucleotides inserted between joined segments). Fig 5D–5F compares more than 2000 LSR unique direct junctions characterized in this study (≈ 13% Sμ-3’RR1 and ≈ 87% Sμ-3’RR2 junctions) to more than 2000 independent unique Sμ-Sγ1 junctions obtained from tonsil. LSR to both 3’RR1 and 3’RR2 significantly differed from CSR with regards to the distribution of the various types of junctions (chi-2 test, p< 0.0001) **(**Fig 5F**)**, notably featuring a much lower frequency of blunt junctions than CSR (7.8% *vs*. 19%), more frequent presence of microhomology and an increased mean length of microhomology (4.1bp *vs*. 2.6 bp). This increased usage of microhomologies was not correlated with a preexisting higher identity between Sμ and the 3’RRs than between Sμ and Sγ (S2 Fig). Insertions, when present in-between joined segments, also showed an increased mean length (4.9bp *vs*. 3.2 bp).

Globally, these data thus show highly diversified Sμ-3’RR DNA junctions occurring in an AID-dependent manner in activated human B-cells.

## Discussion

LSR was previously described in the mouse as a process that deletes all functional Ig genes, thereby terminating B cell function. Like CSR, LSR in mouse B-cells targets a transcribed 3’RR region including abundant DNA repeats shown to bind both Polymerase II and AID in chromatin immunoprecipitation experiments [4,24]. These analogies with CSR suggested that LSR is an additional AID-mediated process participating in the control of B-cell fate. By contrast, in pathology, rare “off-target” lesions mediated by AID can promote chromosome aberrations and B-cell lymphomagenesis. To clarify the potential physiological significance of LSR, we asked if LSR is evolutionarily conserved by examining the ability of the human IgH locus to eventually undergo LSR as described in the mouse and evaluating the frequency and diversity of such recombination events. Analyzing the structure of human IgH 3’RR elements in detail in fact shows their high architectural similarity with the mouse 3’RR. Especially notable are the presence of a central palindrome centered on the hs1,2 enhancer and of several stretches of repetitive LS regions. The human repetitive LS regions are each 0.5 to 2 kb long, longer than LS regions in the mouse 3’RR and approaching the length of the human Sα2 region, with the longest LS flanking the hs4 core enhancer. The conservation of genomic structure of 3’RR sequences suggests that the 3’RR region may be under selection for accessibility to recombination that occurs during the course of CSR to terminate B cell function.

Human and murine 3’RR regions share a further similarity with mammalian S regions. In S regions, the non-template strand is G-rich, promoting R-loop formation and G4 DNA formation [18,19,25]. In S regions, G4-DNA promotes the occurrence of DNA nicks and further excision by exonuclease 1, while pharmacological agents binding G4-DNA can inhibit CSR [18,26,27]. I*n vitro*, G4-DNA was also shown to provide a type of structured DNA which favors the activity of Activation-Induced Deaminase (AID) [19]. Finally, G-quadruplexes forming in S region transcripts were shown to recruit the helicase Ddx1 and contribute to the formation of R-loops before CSR [28]. Using the G4-hunter algorithm, we identified G4-DNA forming sequences within the human 3’RRs together with long G-rich stretches, although in lower amounts compared to S-regions and not restricted to a single DNA strand. Regarding the lack of a preferential orientation, it should be noted that human 3’RR transcription occurs in both the sense and antisense direction (relative to *IgH* constant genes), yielding non-coding eRNA transcripts for which definition of a “template strand” is more ambiguous than for *IgH* constant gene coding regions. In such settings, G4 structures from either DNA strand might recruit AID activity within the 3’RR LS regions, similarly to G4-DNA from the non-template strand within S-regions. It can also be noted that transcription concerns not only eRNA for core enhancer elements but extends throughout the 3’RR, providing another analogy with S regions. The human genome has thus evolved in such a way as to accumulate transcribed DNA repeats, AID target sites and G4 DNA in the vicinity of the 3’RR core enhancers.

In addition to documenting switch-like features in the human 3’RR, we tracked Sμ to 3’RR recombination in human B-cells. Compared to CSR, evaluation of LSR is inherently hindered by its occurrence in suicidal B-cells vanishing within hours while long-term surviving switched cells are stably and readily detectable. Evaluation of the process thus needs to be sensitive and allow full validation of the data by identifying individual sequences of junctions. While such a sequencing approach first requires nested PCR over very large regions and might be jeopardized by amplification biases, this study succeeded in optimizing a both sensitive and semi-quantitative LSR-seq protocol. LSR-seq first confirmed AID-dependence of LSR, and junctions were virtually absent in cells from AID-deficient patients. LSR-seq identified Sμ-3’RR junctions in activated B-cell DNA samples from healthy individuals, with frequencies up to 3% of the detection rate for CSR junctions in similar conditions. Therefore, LSR is not a rare event and it is readily detected in human primary B-cells upon either *in vivo* or *in vitro* activation, demonstrating inter-species conservation of the process first observed in the mouse.

LSR-seq yielded the strongest signals from lymphoid tissues exposed to chronic immune stimulation, such as tonsils and adenoids which contain a mix of naive cells, memory cells (about 20%), abundant GC B-cells with an ongoing activation phenotype (CD20^high^, BCR^low^, CD38+), as well as plasmablasts involved in ongoing local immune reactions to mucosal antigens [29,30]. Studies of samples from various origins also indicated that human LSR junctions were mostly detected transiently, when cells were activated, but not preserved in the long term in quiescent memory B-cells or plasma cells. Notably, peripheral blood includes one-third of memory B-cells in adults, most of them being healthy and long-lived class-switched cells [29]. DNA from blood B-cells accordingly gave high CSRseq signals, as did DNA from bone marrow plasma cells, but low or negative LSR signals. That IgH alleles with LSR deletions are exquisitely rare in memory cells indicates that LSR rarely occurs on the non-functional allele but predominantly targets the functional IgH allele or is biallelic, and therefore incompatible with further survival of B-cells in the absence of a functional BCR. Given the lack of accumulation in memory B-cells (in contrast to CSR), LSR is only transiently detectable concomitantly to *in vivo* B-cell activation. These features fit much better with a programmed suicide rather than with a randomly occurring rare gene alteration, which would then likely occur at similar rates on the functional and the non-functional IgH allele.

LSR junctions were found both in cells activated *in vitro* and in cells activated *in vivo* after CD40 ligation. It was not possible to identify conditions stimulating CSR in the absence of LSR. Even if variations appeared, with a tendency to increase the LSR/CSR ratio when TLR ligands were associated with CD40 ligation, or to decrease this ratio when BCR cross-linking was associated, these variations remained modest. Whether programmed death by LSR is regulated *in vivo* and helps select the best class-switched memory B-cells during Ag-specific responses remains to be demonstrated. While loss or aberration of Ig secretion was shown by Nussenzweig and colleagues in apoptotic GC B-cells and seemed to be AID-dependent [1], it also remains to be shown how different pathways of “Ig production suicide” proceed in such cases (Ig misfolding, stop codons or deletions initiated in the VH region, LSR…) and to define the relative role of each of these processes.

Molecular analysis of human LSR junctions assigned them to either 3’RR1 or 3’RR2, with a distribution showing highly diversified breakpoints. Concerning the donor break, the apparent higher involvement of the most 5’ portion of Sμ likely relates to the preferential amplification of short fragments in a nested PCR assay. Acceptor sites within the large 3’RRs, appear to cluster in three sub-regions each spanning 3 to 5 kilobases, but these sub-regions in fact precede the three unique non-repetitive locations where highly specific reverse primers could be adequately designed for the LSR-seq assay. Because the use of additional regularly spaced reverse primers is prevented by the highly repetitive 3’RR sequence, it is thus possible that the frequency of breaks occurring at larger distances from our primers is under-estimated.

Interestingly, sequence analysis of LSR-seq data from both *in vivo* and *in vitro* activated B-cells revealed the presence of both direct junctions from Sμ to one or the other 3’RR, as well as complex junctions including Sγ or Sα regions in addition to Sμ, indicating that LSR had taken place in previously class-switched cells in at least 14% of cases.

When comparing the sequences of DNA junctions, Sμ breaks appeared highly similar in both CSR and LSR junctions, strongly suggesting identical targeting of Sμ in both processes. Another issue concerns the mechanisms of DNA breakage in the 3’RR which, as for an S region, is repetitive, G-rich and transcribed. While our analysis found that the distance from breaks to AID motifs or G4 DNA was higher for the 3’RR than for S regions, this increased distance likely relates to the less densely repeated AID hotspots and G4 DNA within the 3’RR than within S regions. Nevertheless, observed 3’RR DSBs occurred significantly closer to these hotspots than would be expected if they were randomly distributed. This, in addition to the absence of LSR in AID-deficient patients, confirms active targeting of the 3’RR by AID (even if at a lower level than for Sμ or Sγ). There is, by contrast, no evidence for the implication of enzymes other than AID in LSR breaks, and notably no correlation with positions of APOBEC3 family hotspots.

AID-dependence of LSR, however, does not exclude that, compared to classical Sμ breaks, AID targeting of the 3’RR might occur in a different configuration or with different partners yet to be identified. This could contribute to the increased distance to AID sites and to the occurrence of breaks both within and outside LS repeats. This is reminiscent of the observation of DNA cleavage outside a repetitive target sequence during CSR to the Cδ gene, where the target Σδ sequence harbors few AID hotspots [31]. Experimentally monitoring AID breaks throughout the genome also showed a focus on transcribed loci and to AID consensus sites but without significant involvement of repeats [32]. Even in classical S regions, once initiated in a repetitive region, R-loops eventually extend over distance through nearby G-rich regions, and thereby extend the region targeted by CSR [25]. With a lower density of AID sites in the 3’RR than in Sμ, DSBs might less easily follow cytidine deamination and staggered single strand breaks. More complex processing of SSBs or DNA remodeling on longer distances than within Sμ might then be required before occurrence of a 3’RR DSB, synapsis to an S region and repair. The hypothesis of a change in the structure of staggered broken ends fits with the observed increased length of microhomologies in LSR junctions compared to CSR, and the lower proportion of blunt junctions typical of non-homologous end-joining. Together with the presence of longer insertions, these changes indicate increased involvement of alternate end-joining and microhomology mediated repair.

**Fig 6:**
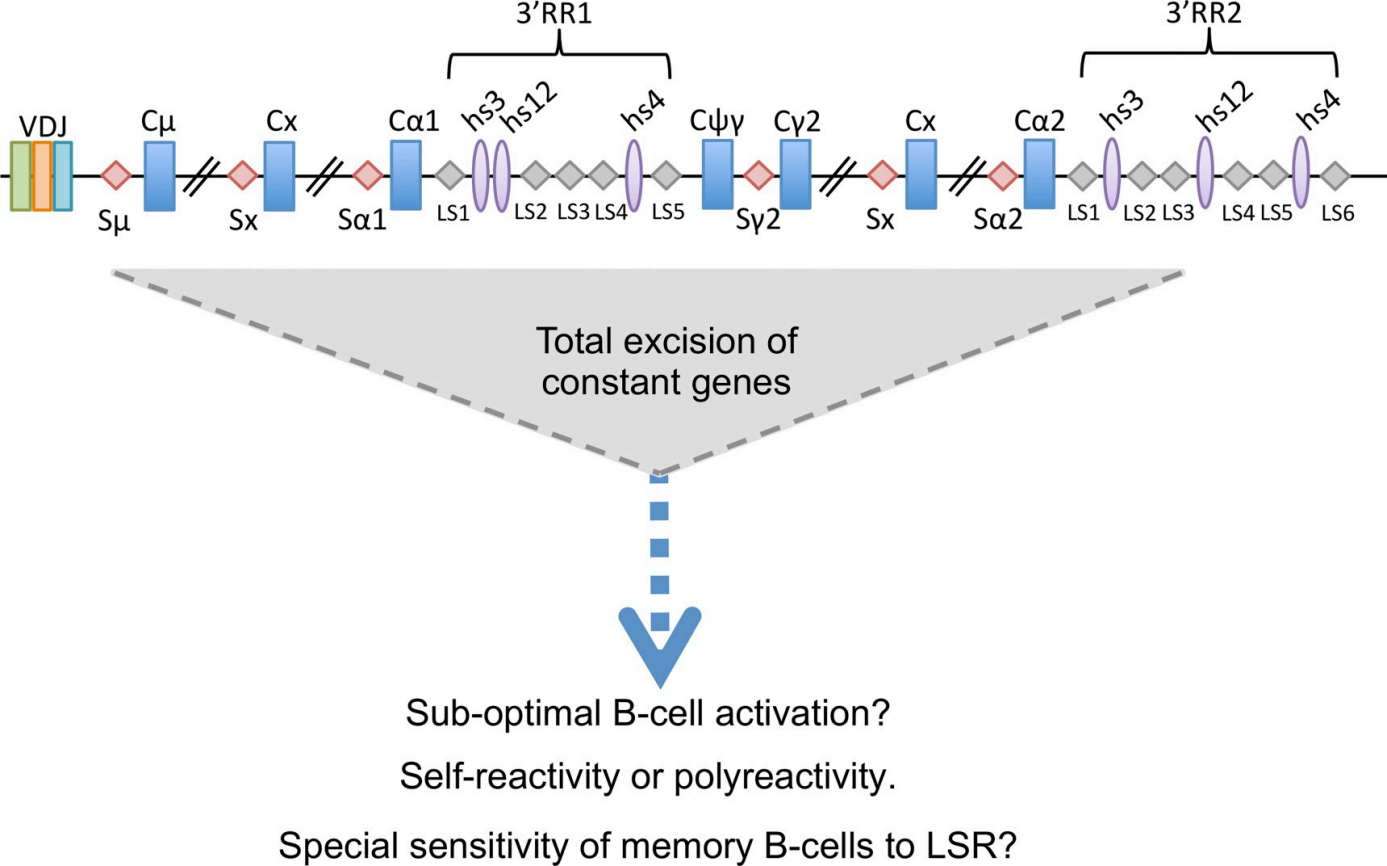
Model of LSR in the human IgH locus. Recombination between Sμ and LS regions in the 3’RR2 super-enhancer results in complete deletion of constant regions of all immunoglobulin classes leading to absence of BCR membrane expression. LSR could be a physiological mechanism to eliminate sub-optimally activated B-cells or those that are poly or autoreactive. Resting memory B-cells seem especially sensitive, as they do not carry stigmas of LSR, which only occurs in activated B-cells.

AID-initiated locus suicide recombination of the IgH locus thus actively occurs in human B-cells, with downstream breakpoints throughout the 3’RR super-enhancers. (Fig 6). The phenomenon either strongly predominates on the functional IgH allele or is bi-allelic, since LSR deletions are mostly detected during B-cell activation but do not remain detectable in memory cells or plasma cells. Because LSR is a transient process towards cell death, it remains difficult to evaluate its regulation and impact on B-cell homeostasis, relative to the other cell death pathways active in the GC. Few methods are available for studying deletions of hundreds of kilobases with loosely determined breakpoints and the use of PCR followed by next generation sequencing remains semi-quantitative, given the PCR obstacles introduced by the repetitive nature of the 3’RR. In such settings, all conditions assayed for B-cell activation induce LSR to some degree and new methods will be necessary in order to determine where and when LSR occurs *in vivo* at the single-cell level.

## Materials and methods

### Ethics statement

Blood from healthy volunteers was collected, after obtaining written informed consent according to the Declaration of Helsinki, from the CHU Dupuytren Hospital, Limoges, France (After approval by the “Comité d’éthique et de protection des personnes du Sud-Ouest”, under the approval number CPP12-012 / 2012-A00630-43) or from the Etablissement Français du Sang (Ethics Committee of CHU Rennes, Research approval # AC-2014-2315). Blood from septic patients was collected with oral informed consent from the Hematology department, CHU Dupuytren. The study was accepted by the Ethics Committee of CHU Dupuytren (Approval number # 184-2015-21). Tonsils were obtained from patients undergoing tonsillectomy performed in CHU of Rennes, while adenoids were obtained from CHU Dupuytren. All were operative pieces and formal written informed consent was not required for the use of entirely anonymized data from which the individual cannot be identified, as indicated by the French Law MR-003 (https://www.legifrance.gouv.fr/affichTexte.do;jsessionid=B1E9EBD60DEB1AA4703C2F7C3CF01D9E.tplgfr26s_3?cidTexte=JORFTEXT000037187443&dateTexte=&oldAction=rechJO&categorieLien=id&idJO=JORFCONT000037186583). Samples from patients with immune deficiencies were obtained with oral informed consent under institutional review board approval by Comité Consultatif de Protection des Personnes participant à une Recherche Biomédicale enregistrée (CCPPRB # 97332, CHU Paris St Antoine, under approval number 1997–0807). Bone marrow aspirates were obtained with written informed consent according to the Declaration of Helsinki from patients undergoing cardiac surgery and recruited under institutional review board approval (Ethics committee “Comité de Protection des Personnes de Rennes”, Approval number # 06/01-567).

### Activation of human B-lymphocytes *in vitro*

Human PBMC were obtained by density gradient centrifugation and human B-cells were negatively sorted from PBMC using EasySep Human B-cell Isolation Kit (StemCell) according to the manufacturer’s instructions. Purified B-lymphocytes were seeded at 1 × 10^6^ cells/ml in IMDM medium (Lonza) supplemented with 20% fetal bovine serum (Deutscher), 1% penicillin/streptomycin (Gibco) and stimulated for 4 days with 100 ng/ml human recombinant CD40L (Enzo Life Sciences) alone or with 50 ng/ml recombinant human IL-4 (Peprotech) or 2 μg/ml Gardiquimod (InvivoGen) or 2.5 μg/ml CpG oligodeoxynucleotide 2006 (InvivoGen) or 100 ng/ml IL-21 (R&D Systems) or 100ng/ml INFγ (R&D Systems) or 50 ng/ml Pam3CSK4 (InvivoGen) or 0.5 μg/ml goat anti-human kappa (Southern Biotech).

### Sorting of naive B-cells, and generation of *in vitro* activated plasmablasts from cultured naive B-cells

PBMCs from healthy volunteers were obtained after density centrifugation. Naive B-cells (NBCs) were purified by negative selection using magnetic cell separation (Naive B-cell Isolation Kit II; Miltenyi Biotec), following the manufacturer’s instructions, with the AutoMACS deplete-sensitive program. Purity of isolated CD19+CD27− NBCs was routinely >99%. Purified NBCs were labeled with 1 μM CFSE (Thermofisher) at 37°C for 10 min and washed in complete medium consisting of RPMI 1640 supplemented with 10% FCS and antibiotics (all from Gibco, Thermofisher). Purified human NBCs were cultured at 7.5 × 10^5^ cells/ml in 24-well plates and stimulated during 4 days with 2.6 μg/ml F(ab′)2 goat anti-human IgA+IgG+IgM (H+L) (Jackson ImmunoResearch Laboratories, West Grove, PA), 100 ng/ml recombinant human soluble CD40L (NCI), 2 μg/ml CpG oligodeoxynucleotide 2006 (Miltenyi Biotec), and 50 U/ml recombinant IL-2 (SARL Pharmaxie). Day-4 activated B-cells were washed and cultured at 4 x 10^5^ cells/ml with 50 U/ml IL2, 12.5 ng/ml IL-10 and 5 ng/ml IL-4 (R&D Systems). At day-6, cells were separated by cell sorting (FACSAria cell sorter, BD Biosciences) into undifferentiated CFSE^hi^ CD38^lo^ (bystander lymphocytes, without proliferation), CFSE^lo^ CD38^lo^ (proliferating activated B-cells), and differentiated CFSE^lo^ CD38^hi^ (plasmablasts) subsets[33].

### Sorting of *in vivo* differentiated early plasmablasts

Actively differentiating plasmablasts from tonsils were purified as previously described [22] as CD19+IgD-CD10-CD38^hi^ cells. CD38^hi^/CD138+ mature plasma cells were purified from bone marrow aspirates.

### RNAseq on activated human B-cells

Human naive B-cells were purified and activated *in vitro* as described (Hipp et al. Nat Commun. 2017). RNA extractions were performed on day 4 IL-2 primed cells with the NucleoSpin RNA XS kit (Macherey-Nagel). Libraries were prepared with the TruSeq Stranded mRNA Library Prep Kit (Illumina) and samples were sequenced on an Illumina NextSeq 500 using 75-bp single-end reads (NextSeq 500 High Output v2, Illumina) by Helixio (Clermont Ferrand, France). Quality of sequencing data was monitored by FastQC. Residual adapters from sequencing were trimmed using Cutadapt 1.0. Potential PCR duplicates were removed using SAMtools 1.3. Reads were then aligned on the GRCh38 human genome using STAR 2.4.2a.

### Amplification of Sμ/Sγ for CSR-seq experiments

DNA from B-cells was extracted using GenElute Mammalian Genomic DNA Miniprep Kit (Sigma). Sμ/Sγ junctions were amplified in triplicate by nested PCR with 200 ng DNA (Herculase II Fusion DNA Polymerase, Agilent Genomics) using the following consensus primers which amplify all four classes of human IgG junctions: PCR1: Sμ1a 5’- CCAGGTAGTGGAGGGTGGTA-3’ and IgGa consensus reverse 5’- GGTCACCACGCTGCTGAG-3’. Amplification was performed according to the following touch down conditions: 98°C for 30 sec; 2 cycles at 98°C for 10 sec, 64°C for 30 sec, and 68°C for 4 min; 3 cycles at 98°C for 10 sec, 62°C for 30 sec, and 68°C for 4 min; followed by 25 cycles at 98°C for 10 sec, 60°C for 30 sec and 68°C, with a final step at 68°C for 5 min. Primers for PCR 2 were as follows: Sμ1b forward 5’- CAGGGAACTGGGGTATCAAG-3’ and IgGb consensus reverse: 5’- CTTGACCAGGCAGCCCAG-3’, according to the following protocol: 98°C for 30 sec, and 30 cycles at 98°C for 10 sec, 60°C for 30 sec, 68°C for 4 min, with a final step at 68°C for 5 min. Forward primers used in both PCRs were located in Sμ while the reverse primers were located in the IgG consensus sequence of the CH1 region.

### Amplification of Sμ/3’RR junctions for LSR-seq experiments

Sμ/3’RR junctions were amplified under the same conditions as for Sμ/Sγ junctions using different primers located at different positions within the 3’ RR. The forward primer located in the Eμ region (EμH1) was used with a reverse primer located downstream of hs4 (3’farhhs4 reverse) or with a primer located in hs1.2 (hs1.2 reverse1) or in hs3 (hs3 reverse 1). PCR1 product was used to perform PCR2 using Sμ1b forward with all reverse primers located in 3’ RR. The primer sequences and PCR programs are given in Table 1.

**Table 1:** Primers and PCR programs used in nested PCR for amplification of LSR junctions.

PCR 1 Primers	Sequence	PCR program
**EμH1 forward**	5’- AGGCTGACCGAAACTGAAAA-3’	
3’farhhs4 reverse	5’- CAAGCGTCAAGGTGTGGAC-3’	98°C for 30 sec; 2 cycles at 98°C for 10 sec, 64°C for 30 s, and 68°C for 4 min; 3 cycles at 98°C for 10 sec, 62°C for 30 sec, and 68°C for 4 min; and 25 cycles at 98°C for 10 sec, 60°C for 30 s and 68°C, with a final step at 68°C for 5 min.
hs1,2 reverse 1	5’-TTCCCAGGGGTCCTGTGGGTC-3’	
hs3 reverse 2	5’- CTGGCCAGGTCTCGGTTTT-3’	98°C for 30 sec; 30 cycles of 98°C for 10 sec, 56°C for 30 sec, and 68°C for 4 min, with a final step at 68°C for 5 min.
**PCR2 primers**	**Sequence**	**PCR programs**
**S**μ**1b forward**	5’-CAGGGAACTGGGGTATCAAG-3’	
Probe 3’hhs4 reverse	5’-GGACGCGGTTTGCTTTTAT-3’	98°C for 30 sec; 30 cycles of 98°C for 10 sec, 58°C for 30 sec, and 68°C for 4 min, with a final step at 68°C for 5 min.
hs1,2 reverse 2	5’-GCTGAGTTTTCGGCATCTCT-3’	98°C for 30 sec; 30 cycles of 98°C for 10 sec, 60°C for 30 sec, and 68°C for 4 min, with a final step at 68°C for 5 min.
hs3 reverse 1	5’-GTTTTGGGGCATGTTTCTCA-3’	98°C for 30 sec; 30 cycles of 98°C for 10 sec, 57°C for 30 sec, and 68°C for 4 min, with a final step at 68°C for 5 min.

### Ion torrent next generation sequencing NGS

Barcoded libraries with 200-bp read lengths were prepared using Ion Xpress plus Fragment Library Kit (Thermo Fisher Scientific) according to manufacturer’s instructions. Each library was prepared using 200 ng PCR2 product. For LSR junction sequencing equal amounts of each PCR2 product (using hs3, hs12 and h4 primers) were mixed together and diluted to 100 pM to prepare one barcoded library. Libraries were run on an Ion PI v3 chip on the Ion Proton sequencer (Life Technologies). Data analysis was performed using CSReport [21].

### Data analysis

Data was analyzed using GraphPad Prism software. Data are shown as means ± SEM of the indicated number of values. The student T test was used to determine significance between stimulation conditions.

## Supporting information

S1 Fig Effect of dilution on number of LSR junctions in activated B cells. A mix of DNA (total 200ng per condition) including **s**uccessively decreasing amounts of tonsil B cell DNA (from 100% to 0%) mixed with T-cell (Jurkat) DNA was used as input for LSR-seq. LSR junctions were amplified with an Sμ forward primer and hs3, hs1,2 and hs4 reverse primers.
(TIF)

S2 Fig Comparison of homologies between Sμ, Sγ1 and 3’RR sequences. Dot plot comparison scoring homologies between Sμ and the three main regions of both 3’RR where LSR occurs close to the hs3, hs1,2 and hs4 core enhancers. Each of the 3 regions assayed was 3kb long and repetitive regions LS1, LS2, LS3 are thus included. Each dot stands for over 70% homology within a 20bp window. Mean identity for each comparison is indicated over each graph. Sμ sequence is on the horizontal axis, 3’RR sequences on the vertical axis. Sμ and Sγ1 for CSR are shown for comparison.
(TIF)
