# Supplementary figures and images for: Locus suicide recombination actively occurs on the functionally rearranged IgH allele in B-cells from inflamed human lymphoid tissues
Source: PLoS Genet. 2019 Jun 14;15(6):e1007721. doi: 10.1371/journal.pgen.1007721 (PMC6594652; doi:10.1371/journal.pgen.1007721)

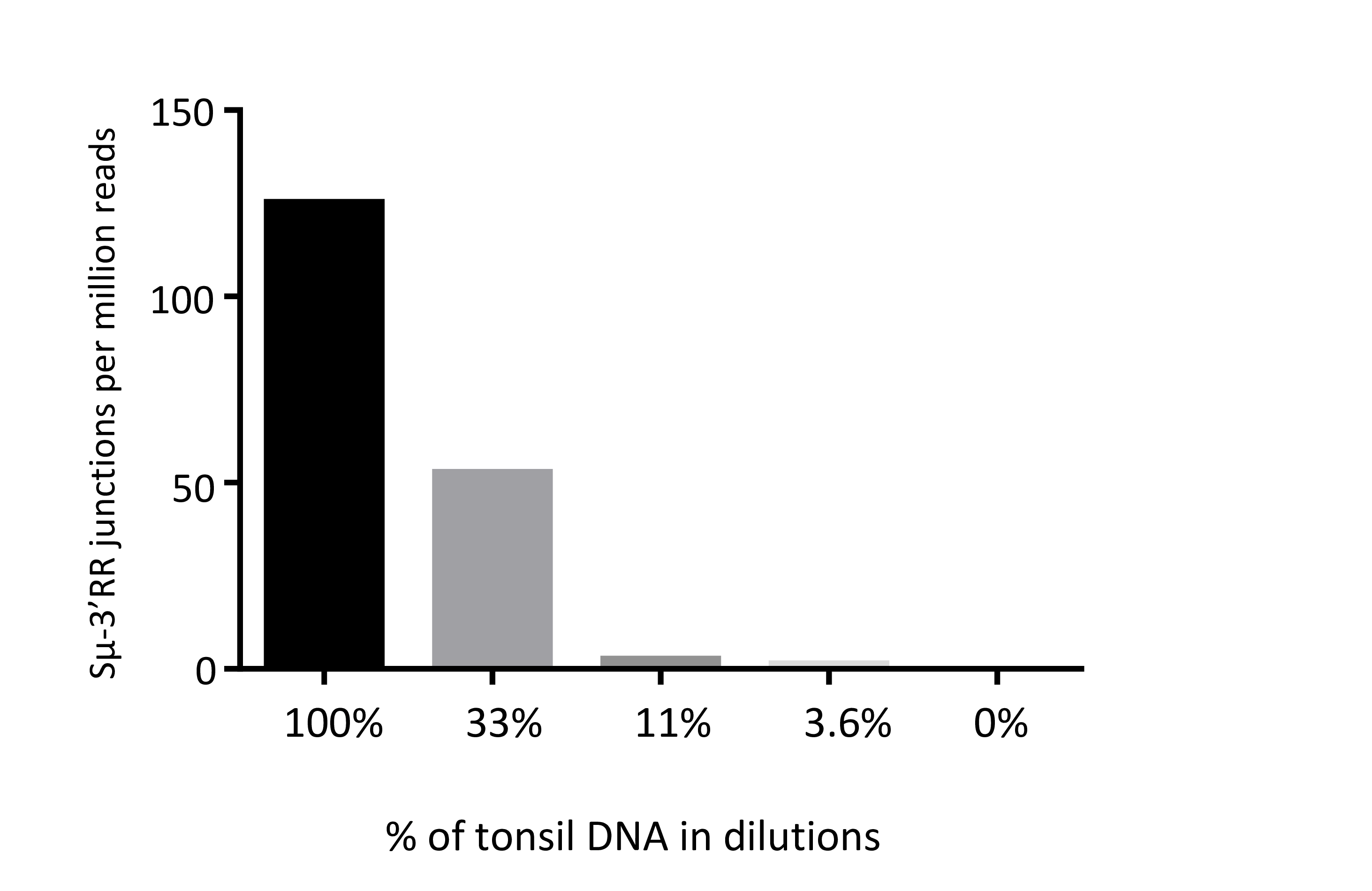

Supplement: S1 Fig — A mix of DNA (total 200ng per condition) including successively decreasing amounts of tonsil B cell DNA (from 100% to 0%) mixed with T-cell (Jurkat) DNA was used as input for LSR-seq. LSR junctions were amplified with an Sμ forward primer and hs3, hs1,2 and hs4 reverse primers. (TIF) [file pgen.1007721.s001.tif]

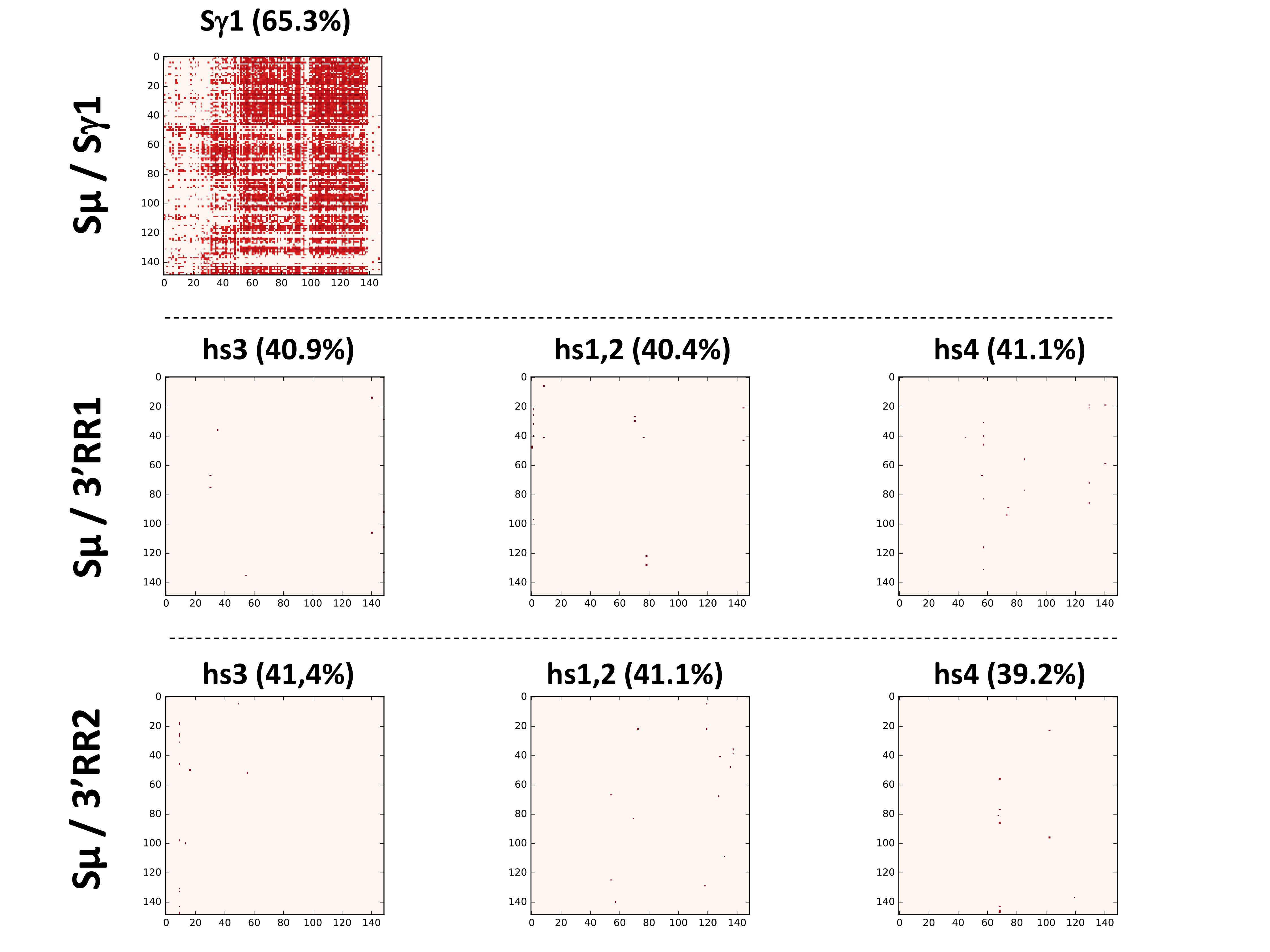

Supplement: S2 Fig — Dot plot comparison scoring homologies between Sμ and the three main regions of both 3’RR where LSR occurs close to the hs3, hs1,2 and hs4 core enhancers. Each of the 3 regions assayed was 3kb long and repetitive regions LS1, LS2, LS3 are thus included. Each dot stands for over 70% homology within a 20bp window. Mean identity for each comparison is indicated over each graph. Sμ sequence is on the horizontal axis, 3’RR sequences on the vertical axis. Sμ and Sγ1 for CSR are shown for comparison. (TIF) [file pgen.1007721.s002.tif]
